# Prospects for improvement of coffee quality and stress resilience using genomic and genetic resources

**DOI:** 10.3389/fpls.2026.1966236

**Published:** 2026-10-06

**Authors:** Mohan Singh Rajkumar, David A. Laughlin, Fabián Echeverría-Beirute, Roger D. Norton, Kranthi K. Mandadi

**Affiliations:** 1 Texas A&M AgriLife Research and Extension Center, Weslaco, TX, United States; 2 Programa de Doctorado en Ciencias Naturales para el Desarrollo (DOCINADE), UNA, ITCR, UNED, Heredia, Costa Rica; 3 Center for Coffee Research and Education, Borlaug Institute for International Agriculture and Development, Texas A&M University, College Station, TX, United States; 4 Department of Plant Pathology and Microbiology, Texas A&M University, College Station, TX, United States; 5 Institute for Advancing Health Through Agriculture, Texas A&M AgriLife, College Station, TX, United States

**Keywords:** coffee, coffee-quality, genetic diversity, genomics, multi-omics, stress resilience

## Abstract

Coffee is one of the most widely traded and highly valuable commodities, known for its pleasant flavor, unique aroma, and caffeine’s stimulatory effect. Beyond their sensory perceptions, coffee beans are rich in several bioactive compounds and biomolecules beneficial to human health. Only two species, *Coffea arabica* and *Coffea canephora*, account for nearly all the world’s coffee production, owing to their high quality and commercial value. Despite low variability and narrow genetic diversity, coffee plant genomic resources are underrepresented. Recent advancements in high-throughput sequencing technologies and reduced costs have enabled the development of various crop genomic resources, including coffee. In this review, we systematically cataloged the genetic and genomic resources available to the coffee research community, including reference genomes, DNA polymorphisms, transcriptomes, and non-coding regulatory transcripts. Furthermore, we highlighted promising candidate genes that may have functional roles in improving coffee quality, yield, and stress resilience. These resources provide an excellent knowledge base for studying genetic factors influencing coffee quality and agronomic traits, and for developing new breeding and CRISPR-based biotechnological strategies for coffee improvement.

## 1 Introduction

Coffee plants (*Coffea* spp.) are perennial shrubs belonging to the *Rubiaceae* family. They are an essential source of several therapeutically beneficial secondary metabolites, including caffeine, chlorogenic acid, and caffeic acid (Ding et al., 2023). *Coffea spp* comprises ~139 species (Guyot et al., 2020; Salojärvi et al., 2024). However, *Coffea arabica* (*C. arabica*) accounts for ~60% of global coffee production and is cultivated in more than 70 countries, followed by ~40% by *Coffea canephora* (*C. canephora*), also known as *Coffea robusta* (*C. robusta*) (Tran et al., 2016). Worldwide, ~125 million people are engaged in coffee production and allied industries (Tsigkou et al., 2025).

Most *Coffea* spp. are diploid and self-incompatible, which generally promotes outcrossing. However, the low genetic diversity and bottleneck effect among the cultivated coffee are likely the consequences of biased artificial selection rather than the direct effect of self-incompatibility itself (Salojärvi et al., 2024). *C. arabica* is the only allotetraploid derived from natural crossbreeding between paternal *C. canephora* and maternal *Coffea eugenioides* (*C. eugenioides*) (**Figure 1**; created with BioRender.com) and is capable of self-fertilization, unlike its diploid parents (Lashermes et al., 1999). The optimal growing conditions for *C. arabica* are tropical highlands. In contrast, *C. robusta* is better adapted to lower altitudes and warm temperatures. Adapting the coffee species to different environmental conditions affects coffee quality. However, the detailed physiological and molecular mechanisms underlying coffee’s geographical adaptation, as well as the factors shaping flavors, remain largely unexplored. For example, the introduction of wild *C. stenophylla* in West Africa is suggested as a valuable genetic resource for coffee improvement due to its adaptation to a wide range of temperatures and rainfall conditions, making it of interest to breeding programs aimed at climate resilience while preserving beverage quality like in *C. arabica* (Davis et al., 2021). *C. stenophylla* is of particular interest as a wild genetic resource. It offers a potential reservoir for broadening the genetic base and increasing allele diversity to improve agronomic traits in coffee. Analysis of genetic resources can reveal molecular mechanisms and key genes for the development of new varieties and hybrids.

**Figure 1:**
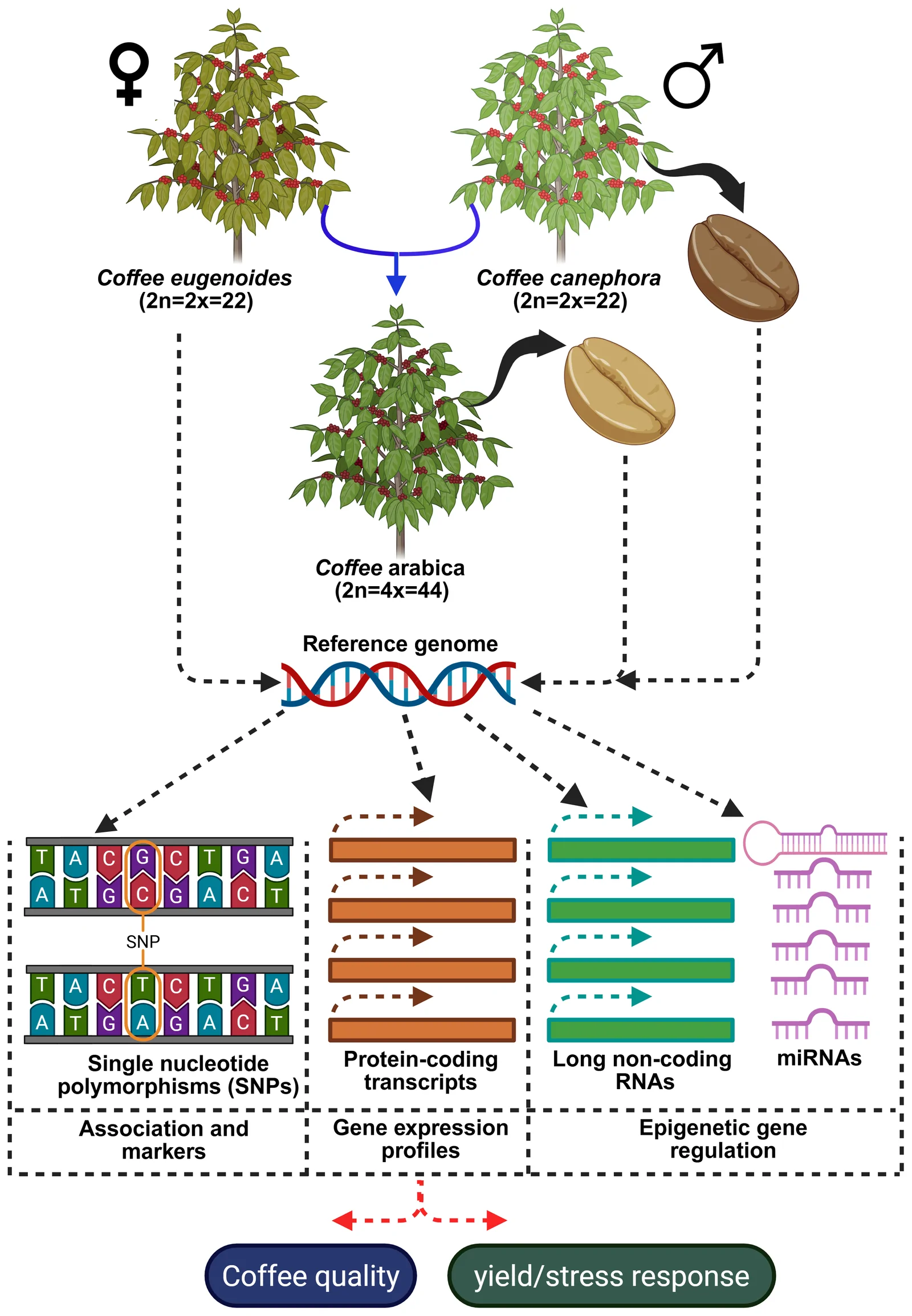
An integrative multi-omics approach to improve coffee variety with high yield and superior quality. A comprehensive data collection of genomes, epigenomes, transcriptomes, and metabolomes from diverse coffee accessions, and a strategy of integrative analysis to identify regulatory networks, mechanistic insights, and prioritize candidate genes, followed by functional validation using biotechnological applications for improving coffee quality and yield, and enhancing resilience to abiotic stress and defense response, is presented.

Although *C. arabica* accounts for ~60% of coffee production, it is more vulnerable to multiple biotic and abiotic stresses than its ancestral progenitors and other wild relatives (DaMatta et al., 2007; Salojärvi et al., 2024). For instance, several coffee cultivars are known to be sensitive to drought (Mofatto et al., 2016; Santos et al., 2015; Silva et al., 2022), heat (Marques et al., 2021, Marques et al., 2024; Yamane et al., 2022), salinity stresses (de Lima et al., 2014), and are susceptible to a wide range of pathogens (Lu et al., 2022; Peck and Boa, 2023; Waller and Masaba, 2006). Introgression of cultivated coffee with wild relatives, followed by selection of progenies resilient to biotic and abiotic stresses without affecting coffee quality, could increase yield and quality, and expand the repertoire of coffee germplasm and natural variation. For example, the culmination of international collaboration of PROMECAFE in the 90s led to the development of F1 hybrids (Centroamericano) with desirable agronomic traits, such as higher yields, enhanced disease resilience, and better coffee cup quality (McCook and Montero, 2024).

Despite environmental factors playing crucial roles in the adaptation and development of coffee, the role of genotype-by-environment interactions remains largely unexplored. This knowledge gap is particularly important under climate change scenarios, where cultivating coffee accessions adapted to specific environmental conditions would be necessary. In addition, trials of coffee cultivation in multi-environmental conditions across varying altitudes, fluctuating temperatures, and water availability would be essential to assess interactions of genotypes and environmental conditions. Together with genomic approaches, the development of resilient coffee varieties adapted to challenging multi-environmental conditions would be increasingly necessary. Nevertheless, further exploration and efforts are needed to broaden the genetic base of coffee varieties/hybrids to enhance the crop’s resilience to the challenges posed by rising heat, drought, and disease, while preserving cup quality and yield. In this review, we cataloged recent advances in coffee omics resources (**Figure 1**) and identified their utility for coffee improvement and research.

## 2 Coffee genomes and polymorphisms

Efforts have been made to draft high-quality genomes of coffee species, especially *C. canephora, C. eugenioides*, and *C. arabica.* The sequencing of *C. canephora* revealed a genome size of ~710 Mb, assembled into 11 chromosomes (2n=2x=22), and annotation of about 25,574 protein-coding genes (PCGs) (Denoeud et al., 2014). Likewise, ~682 Mb of genomic regions, accommodating about 32,192 genes, were detected in *C. eugenioides* (Salojärvi et al., 2024). *C. arabica*, as an allotetraploid derived from crossbreeding *C. canephora* and *C. eugenioides*, has a genome size almost double the size of its parents (2n=4x=44 assembled in 22 chromosomes), spanning 1,320 Mb (Scalabrin et al., 2024) and 1,281 Mb (Salojärvi et al., 2024). In addition, *C. arabica* harbors a much larger number of protein-coding genes (PCGs; ~50,000) than its parents (Medrano et al., 2025; Salojärvi et al., 2024; Scalabrin et al., 2024). *C. arabica*, as an allotetraploid derived from hybridization between *C. canephora* and *C. eugenioides*, possesses two parental subgenomes and homeologous gene copies. Thus, the frequency of duplication and homeologous genes in *C. arabica* is probably high. However, the occurrence of other genetic variations and alternatively spliced transcripts could be selected by other mechanisms distinct from polyploidization. For instance, ~95,995 distinct transcript isoforms were detected in *C. arabica* using PacBio’s ultra-long-read Iso-seq, which is much larger than its ancestral progenitors (Cheng et al., 2018). Notably, the difference in gene numbers among the coffee accessions may not be solely due to differences in genome sizes and ploidy level. Variations in genome assembly quality, gene annotation and prediction algorithms, and transcriptomic evidence could also contribute to differences in gene counts.

Allopolyploidization is widespread in higher plants and is crucial in diversifying and evolving new plant species. An earlier study estimated that the emergence of *C. arabica* occurred between 0.046 and 0.665 million years ago (MYA) based on comparative sequence analysis of *C. arabica* and *C. canephora* across eight orthologous gene pairs. Moreover, insertion of transposons, especially Ty1-copia, substantially contributed to the expansion of the *C. arabica* genome (Yu et al., 2011). However, a recent report suggested that the divergence between ancestral progenitors and wild accessions occurred ~0.3 MYA, and that the polyploidy event in *C. arabica* occurred ~0.35 to 0.61 MYA, indicating a more recent origin of *C. arabica* (Salojärvi et al., 2024). Analyzing the expression of homeologous genes from the two diploid parents in *C. arabica* suggested that biased expression of homeologs may be linked to polymorphisms in cis-regulatory elements within the genetic sequences (Combes et al., 2012).

Whole-genome sequencing (WGS) and Genotyping-by-Sequencing (GBS) of multiple coffee species, cultivars, ecotypes, and populations can identify genetic variants, including single-nucleotide polymorphisms (SNPs) and insertions/deletions (InDels). The two approaches differ mainly in that WGS covers extensive genomic regions but accommodates fewer samples. In contrast, GBS allows multiplexing of numerous samples but covers only selected regions of the genome. SNPs can also provide valuable information for predicting phenotypes of numerous accessions using genomic prediction, thereby substantially reducing the labor, time, and resources required to screen phenotypes across large populations and numerous accessions. For the first time, genomic prediction was implemented in coffee using SNPs derived from the GBS data of *C. canephora* recurrent populations to evaluate individuals with better yield and resistance to leaf rust (Ferrao et al., 2019). Similarly, genetic analysis of *C. canephora* populations collected from West Africa identified phenotypic variations that could better adapt to abiotic stress conditions (Pokou et al., 2025). In *C. arabica*, too, SNPs from 195 individuals were used to predict phenotypic traits related to yield and biotic stress resilience using genomic prediction (Nascimento et al., 2024). Despite coffee’s long juvenile period, genomic prediction can accelerate the evaluation of yield and quality phenotypes in complementation with conventional phenotyping. However, the limited availability of coffee genomic resources hampers the evaluation of phenotypes using genomic prediction. The broader implementation of genomic prediction in coffee depends on the availability of dense genotypic data, phenotypically trained populations, and information on genotype × environment interactions. These resources will be essential for the effective implementation of genomic prediction in coffee breeding programs.

SNPs and InDels are widespread across accessions/species and are unevenly distributed along the genomic regions/chromosomal locations. Moreover, a substantial fraction of DNA polymorphisms is expected to be neutral and not linked to gene functions. Quantitative trait loci (QTLs) are genomic regions that contain genetic polymorphisms (SNPs and InDels) and are statistically associated with variation in a quantitative trait. Genes overlap with QTLs that contain associated SNPs/InDels, which have the potential to modulate differential gene functions. It confers genetic factors for phenotypic plasticity and trait determination across populations, accessions, or species. QTL mapping in coffee is scarce, offering an excellent opportunity to identify the genetic basis of agronomic traits. Association mapping using genome-wide association studies (GWAS) enables the identification of associations with phenotypic variation. A study employed an integrative analysis of metabolite profiling and GWAS across 80 coffee accessions to underscore coffee quality. Three SNP markers most significantly associated with caffeine, trigonelline, and 5-caffeoylquinic acid were identified (Jose Luis et al., 2022). Another study identified quantitative trait nucleotides (QTNs), analogous to significantly associated SNPs, from a panel of 120 *C. arabica* accessions. Four QTNs colocalized with genes, including serine/threonine-kinase and nucleotide-binding site leucine-rich repeat (NBS-LRR), were significantly associated with Bacteria halo blight (BHB) disease (Ariyoshi et al., 2022).

Similarly, WGS of 736 *C. arabica* accessions revealed low genetic diversity, which suggested recent selection and a severe bottleneck, presumably arising from a single polyploidization event (Scalabrin et al., 2020). WGS of 18 coffee accessions differs in caffeine content, identified 65 non-synonymous SNPs significantly associated with caffeine content, 11 of which overlapped with genes encoding enzymes involved in caffeine biosynthesis (Tran et al., 2018). WGS of 90 coffee accessions identified SNPs significantly associated with traits such as caffeine content, yield, and biotic stress response (Mekbib et al., 2022). WGS of 93 coffee accessions identified ~15 million high-frequency SNPs, including two gene families involved in disease resistance (Huang et al., 2020a). Sequencing a selected region of the genomes of 80 coffee accessions using DArTseq (Diversity Arrays Technology Sequencing) also identified at least three significantly associated SNPs with the biosynthesis pathways of caffeine and the plant alkaloid trigonelline, known for health benefits (Jose Luis et al., 2022). Recently, genome sequencing of 174 coffee accessions revealed several chromosomal aberrations, including aneuploidy, deletions, duplications, and nonreciprocal chromosomal exchanges, indicating the incorporation of genetic variation into the homoeologous genome in allotetraploid *C. arabica* (Scalabrin et al., 2024). In the context of allopolyploid species like *C. arabica*, which derived from intercrossing distinct ancestral progenitors, such structural variations can play a crucial role in genome evolution by altering homoeologous chromosome composition, gene dosage, and subgenome balance. Subsequently, they can contribute to environmental adaptation and phenotypic plasticity, including coffee quality traits. Therefore, those structural variations could serve as valuable resources for trait discovery and markers for breeding applications in coffee.

QTL mapping and GWAS are primarily used to identify genomic regions or markers associated with trait variation. QTL mapping typically relies on biparental or structured populations, whereas GWAS exploits natural variation across diverse germplasms. In contrast, genomic prediction uses genetic markers, usually SNPs, to predict phenotypes. The significant genetic marker, validated by its association with traits, can be effectively applied via MAS in breeding populations to select individuals carrying favorable alleles. Together, these approaches are complementary and can be integrated into smart agricultural practices to improve coffee breeding programs.

Recently, the pangenomes of crop plants have garnered attention for their completeness of genomic sequences across different accessions of a species, rather than relying on a single reference genome. In the context of coffee, high-quality reference genomes of only three species, such as *C. canephora*, *C. eugenioides*, and *C. arabica* have been released. Despite coffee’s narrow genetic base and the bottleneck effect, the pangenome approach, integrating multiple coffee accessions, including wild relatives, could reveal novel genes/alleles and structural variants absent from independent reference genomes. Therefore, coffee pangenome resources can be effectively harnessed for gene discovery and marker development associated with agronomic traits and breeding applications.

## 3 Omics pertaining to the coffee quality

Coffee quality is a complex trait influenced by many factors, including genotype, environment, agronomic practices, and developmental stage. The biochemical composition of the green/ripened coffee beans includes alkaloids, phenolics, lipids, sugars, and volatile compounds, which contribute to quality attributes. However, final cup quality is determined not only by the biochemical composition of the coffee beans but also by postharvest processing, storage, and roasting/brewing conditions. Consequently, sensory attributes include aroma and flavor, as well as the balance of acidity, bitterness, sweetness, and aftertaste that determines coffee cup quality. Efforts have been made to identify genes that could be involved in coffee quality via omics approaches. Metabolomic profiling revealed that the endosperm of *C. arabica* is enriched with chlorogenic acid, cell wall polysaccharides, and unusual fatty acids (Joët et al., 2009). A unique type of lipid enriched with diterpenes, the so-called coffee oil in coffee beans, and an abundance of 5-hydroxytryptamine primarily in the outer wax layer of the coffee bean have been reported (Speer and Kölling-Speer, 2006). A type of polysaccharide (galactomannans) represents up to 25% of the mature seeds of coffee beans. The expression of two key enzymes encoding *mannan synthase* (*ManS*) and *galactomannan galactosyl transferase* (*GMGT*) was found to be involved in polysaccharide content in the endosperm cell walls of developing coffee seeds (Pre et al., 2008). Gene regulatory networks (GRNs) for transcripts and metabolites involved in carbohydrate metabolism and the expression of *cell wall storage polysaccharides* (*CWSPs*) in endosperm were identified. Moreover, high expression levels of galactomannan biosynthetic genes were implicated in the cell wall architecture of the coffee seeds (Joet et al., 2014).

The fruit’s skin color usually indicates its maturation stages and can consequently determine the appropriate developmental stage for fruit quality. Transcriptome dynamics during the maturation of coffee beans across three stages: immature (green), yellow (intermediate), and red (mature) *C. arabica* seeds were analyzed. The expression levels of genes encoding *galactomannan*, *triacylglycerol* (*TAG*), *TAG lipase*, and storage proteins were higher at later stages of seed development (Cheng et al., 2018). Integrative analysis of metabolomes and transcriptomes showed co-expression of genes encoding enzymes such as *3AT*, *BZ1*, and *lcyE*, along with *MYB*, *NAC*, and *bHLH* TFs in regulating the biosynthesis of anthocyanins and carotenoids during fruit peel coloration in *C. arabica* (Wang et al., 2024). However, natural variations in fruit color among coffee accessions, due to differences in pigment and metabolite accumulation, could be indirectly linked to coffee quality. Transcriptome analysis of four *C. arabica* accessions differing in mature fruit color (orange, purple, red, and yellow) identified differential expression of a handful of genes involved in carotenoid and anthocyanin biosynthesis. Moreover, high expression of genes encoding *PSY*, *β-CHY*, *CCD7*, *NCED*, and *ABA2* was implicated in yellow fruits in *C. arabica* (Hu et al., 2022).

Previous studies examined the regulation of caffeine biosynthesis in coffee seeds. The genes encoding *N-methyltransferases* (*NMTs*) catalyze caffeine biosynthesis by N-methylating and removing ribose moieties from the precursor xanthosine. A study demonstrated that three distinct *CaNMTs* of *C. arabica* (*CaXMT1*, *CaMXMT2*, and *CaDXMT1*) catalyzed the conversion of xanthosine to caffeine in *E. coli* (Uefuji et al., 2003). A transcriptomic study followed by coexpression analysis of coffee beans and tea leaves revealed conserved roles for *NMTs* in catalyzing the conversion of xanthosine to caffeine (Zhang et al., 2022). Another study analyzed the abundance of non-nutritional ingredients, such as phytate and Fe-rich proteins (ferritin), in coffee seeds and their relationship to coffee quality (Nobile et al., 2010). Although coffee fruits usually contain two hemispherical beans, occasionally peaberries, single spherical seeds derived from the fertilization of only one embryo, are often perceived as premium in some coffee markets (Suhandy et al., 2018). Peaberries are often considered or marketed as having distinctive or superior quality. However, consistent experimental evidence demonstrating superior sensory qualities remains limited. A study analyzed the transcriptome dynamics of peaberries versus regular *C. arabica* beans and identified that differentially expressed genes (DEGs) were enriched in processes related to cell wall modification, lipid binding, protein binding, oxidoreductase activity, and transmembrane transport. In addition, a few candidate genes involved in flavonoid biosynthesis were implicated in distinguishing peaberries from regular coffee beans (Fu et al., 2023). A transcriptomic study in the parent *C. eugenoides* revealed dynamic expression of ∼5,000 genes between fruit and leaf tissues, providing a resource for exploring evolutionary perspectives and identifying candidates for agronomic traits (Yuyama et al., 2016), as wild coffees are presumed to harbor stress-resilient alleles (Hollister, 2015; Ramsey, 2011).

Overall, phenotyping coffee quality is challenging due to the involvement of multiple factors and several agronomic attributes. In this context, the assessment of metabolomes and volatile compounds helps quantify the balance among sugars, acids, alkaloids, phenolics, and aroma-related volatiles, which are directly linked to sensory evaluations of coffee cup quality perceived by consumers. The emerging high-throughput phenotyping approaches, together with trials in multi-environmental conditions and multi-omics approaches, will significantly contribute to deciphering the complex trait of coffee quality.

## 4 Omics pertaining to the coffee stress responses

Coffee grown on irrigated farms typically yields higher than that grown on rain-fed farms (Silva et al., 2022). Higher accumulation of metabolites, mainly pheophytin, a crucial component of photosynthetic activity, was observed in irrigated coffee plants, suggesting greater stress in non-irrigated plants, which mimics a drought stress response (Scheel et al., 2019). Transcriptome analysis of *C. arabica* fruit/bean suggested the expression of genes involved in raffinose biosynthesis, indicating modulation of stress-responsive genes encoding osmoprotectants during the initial stages of fruit development (Ivamoto et al., 2017). Two *C. canephora* accessions with contrasting responses to drought stress showed higher expression of genes encoding *galactinol and raffinose family oligosaccharides* (*RFOs*) in the tolerant genotype (Santos et al., 2015). In *C. arabica*, too, three isoforms of the *RFO* gene were identified, and their expression under abiotic stress conditions in leaf tissues was prominent (dos Santos et al., 2011). Another study compared the shoot apex transcriptomes of *C. arabica* cultivars with contrasting responses to drought stress under irrigated versus water-stressed conditions. A few known genes involved in drought tolerance, such as *CaSTK1*, *CaSAMT1*, *CaSLP1*, *CaMAS1*, and *nsLTP*, exhibited higher expression in the drought-stress-tolerant cultivars (Mofatto et al., 2016). Moreover, drought-tolerant coffee progenies showed better adaptation, as evidenced by upregulation of stress-responsive genes encoding *CaMYB1*, *CaERF017*, *CaEDR2*, *CaNCED*, *CaAPX1*, *CaAPX5*, *CaGolS3*, *CaDHN1*, and *CaPYL8a* under drought stress (de Oliveira Santos et al., 2021). In addition, drought-tolerant coffee seeds are better adapted to desiccation stress during storage, as revealed by correlations with reduced physiological activities, including metabolism, photosynthesis, and respiration, and by the activation of stress-responsive genes (Dussert et al., 2018). Similarly, the heat-stressed coffee plants also exhibited reduced catalase (CAT) and superoxide dismutase (SOD) activity and elevated levels of reactive oxygen species (ROS) and peroxides (Yamane et al., 2022). A study showed dynamic expression of genes involved in photosynthesis, including *RUBISCO*, chlorophyll-binding protein, and photosystems I and II, under heat stress and elevated air CO_2_ levels (Marques et al., 2021). Collectively, drought and heat stress in coffee correlated with negative regulation of photosynthesis- and physiology-related genes. In contrast, positive regulation of genes encoding protective proteins, such as dehydrins and heat shock proteins, is evident (Marques et al., 2024). A study comparing leaf transcriptomes of *C. arabica* with its diploid parental species (*C. canephora* and *C. eugenioides*) at increasing temperatures showed that *C. arabica* was more homeostatic, suggesting better adaptability than its parents (Bertrand et al., 2015). Adopting heat-resilient coffee genotypes may be required due to the projection of global warming. The transcriptomes of two *C. arabica* genotypes differing in heat sensitivity in optimal and elevated temperatures were analyzed (de Oliveira et al., 2020). The results showed genotype-specific DEGs, primarily related to carbohydrate metabolism, indicating biomarkers relevant to thermotolerance in a global warming-like scenario.

In addition, biotic stresses are a significant concern in coffee plants. Plant-pathogen interactions are broadly classified into incompatible and compatible. The incompatible interaction is mainly due to the host’s resistance to the pathogens. Conversely, in compatible interactions, the host can mount a limited response to pathogen-induced damage, which can be considered tolerance to the pathogen. A study analyzed responses of thirteen *C. arabica* cultivars differing in susceptibility to coffee leaf rust caused by the fungus *Hemileia vastatrix*, categorizing them as resistant (R), moderately resistant (MR), or susceptible (S). The expression of defense-responsive genes was correlated with disease susceptibility using RT-qPCR analysis (Reichel et al., 2022). Another study employed RNA-seq to identify genome-wide gene expression in response to rust disease progression in susceptible and tolerant *C. arabica* (Florez et al., 2019). The incompatible interaction between the host and pathogen in the tolerant cultivar was correlated with the expression of receptor-like kinases, changes in ion fluxes, ROS production, protein phosphorylation, ethylene biosynthesis, and callose deposition (Florez et al., 2019). Another study identified 743 DEGs in response to root-knot nematode invasion in *C. arabica* and numerous other DEGs across different plant species, indicating evolutionarily conserved genes responding to the nematode (Mota et al., 2020).

Likewise, the expression of coffee genes in response to leaf miner damage caused by *Leucoptera coffella* was analyzed in susceptible and tolerant interspecific hybrids of *C. arabica* and *C. racemosa*. Genes involved in cellular function and maintenance were upregulated in the tolerant cultivar. DEGs between the susceptible and tolerant healthy cultivars implicated molecular insights into coffee plants’ defense response to biotic stress at least at the transcriptional level (Cardoso et al., 2014). In addition, *U-box* genes involved in biotic stress tolerance have been identified, and their expression varied across different organs in *C. canephora* (Liu et al., 2024). Priming chemicals that mimic pathogenic antigens can improve a plant’s defense responses by memorizing the immunogenic response in subsequent infections. The application of hexanoic acid to rust-susceptible and tolerant *C. arabica* cultivars, followed by RNA-seq of control (CT) and hexanoic acid-primed coffee plants, indicated modulation of cellular metabolic processes induced by priming (Budzinski et al., 2022).

## 5 Non-coding RNAs in coffee biology

Most RNAs do not code for functional proteins but play essential roles in gene regulation, a category known as non-coding RNAs (ncRNAs). Mechanistic insights into ncRNA-mediated gene regulation and biological processes remain largely unexplored in coffee. Efforts have been made to deploy in silico computational prediction to identify ncRNAs and expression profiling via smRNA sequencing. The in silico prediction mainly relies on the ability of miRNA precursors to form hairpin structures and sequence homology with other known miRNAs from different plant species. In addition, the miRNA targets are predominantly predicted computationally.

A few studies have deployed in silico approaches for miRNA identification in coffee. The screening of *C. arabica* expressed sequence tags (ESTs) identified a putative miRNA and its target protein-coding genes (PCGs) (Akter et al., 2014). About 20 putative miRNAs relevant to broad-spectrum biological processes in *C. arabica* (Devi et al., 2016), ∼18 miRNAs targeting 41 PCGs in *C. canephora* (Rebijith et al., 2013), and ∼36 miRNAs targeting 616 and 362 PCGs in *C. arabica* and *C. canephora*, respectively (Chaves et al., 2015) were computationally predicted. A study predicted ∼317 miRNAs in *C. canephora* using the plant miRNA database and identified the functional relevance of three miRNAs (miR156, miR172, and miR390), including their roles in structural organization, phylogenetic relationships, and putative target PCGs (Noronha Fernandes-Brum et al., 2017). Similarly, ∼92 miRNA precursors and ∼159 PCG targets in *C. canephora* were computationally predicted (Lemos et al., 2020); several were conserved with *C. arabica* (Loss-Morais et al., 2014). In addition, five genes encoding *Dicer-Like* (*DCL*) involved in miRNA processing and RNA interference (RNAi) were predicted in *C. arabica*, using bioinformatics analysis (Parvez Mosharaf et al., 2019).

A limited study of ncRNA profiling using smRNA sequencing has been conducted. The smRNA sequencing of root tissues of *C. arabica* under control and nitrogen-starvation conditions identified ∼86 miRNAs targeting 253 PCGs (dos Santos et al., 2019). A study profiled small RNAs during the vegetative-to-reproductive transition and floral organ development in *C. arabica*. Most miRNAs accumulate in a stage-specific manner. However, members of the miR482 and miR2118 superfamily originated from genomic loci that colocalized with the resistance genes and exhibited coexpression across developmental stages (Cherubino Ribeiro et al., 2024).

LncRNAs are another class of ncRNAs; they share many similar features with coding transcripts. Therefore, lncRNA expression profiling can be inferred from RNA-seq data. Recently, a study computationally predicted ∼5064 long non-coding RNAs (lncRNAs) from the transcriptome data of *C. canephora*. The lncRNAs were classified into different types based on their orientation/proximity to neighboring PCGs: sense and antisense, intronic, and intergenic (Lemos et al., 2020). Another study in *C. arabica* used RNA-seq data from ∼260 samples representing fruits, leaves, stems, and roots to explore the lncRNA repertoire. A total of ∼10,564 lncRNAs were identified, mostly (77.14%) found in the intergenic regions. Several lncRNAs showed dynamic expression between the fruit and other vegetative organs, including the leaf, stem, and root (Abdel-Salam et al., 2021), suggesting broader roles in coffee’s growth and development. Despite advances in the computational identification of coffee ncRNAs and in expression profiling, their functions through genetic analysis remain uncharacterized, presenting an excellent opportunity for coffee improvement.

## 6 Gene family analysis in coffee for crop improvement

In higher plants, multiple genes (members) with conserved structures and functions are organized into families. They primarily arose from duplication events and integration of transposable elements (TEs), followed by functional and evolutionary divergence. Many coffee gene families that could be involved in development, coffee quality, and stress resilience have been analyzed (**Table 1**). Some of the gene families involved primarily in development include *APETALA2/Ethylene Responsive Factor* (*AP2/ERF*), *MADS-box*, and *expansin* (*EXP*). Although numerous *AP2/ERF* members were identified and conserved across the parental species (*C. canephora* and *C. eugenioides*) and offspring *C. arabica*, a subgroup of the *AP2/ERF* family with extensive variation was suggested to have undergone species-specific expansion (Park et al., 2025). Analysis of *MADS-box* genes to understand flower and fruit development in *C. arabica* verified the expression of a few members in different floral organs using *in situ* hybridization (de Oliveira et al., 2014). Analysis of the expansin (*EXP*) gene family in *C. arabica* cultivars differing in fruit size revealed differences in expression levels of *CaEXPA1* and *CaEXPA3* in the perisperm, which is a crucial tissue responsible for coffee bean size, and expression of *CaEXPA2* in the pericarp. The results suggest they may contribute to coffee yield by regulating fruit size (Budzinski et al., 2011).

**Table 1:** Gene families of coffee species involved in diverse biological functions, including development, somatic embryogenesis for biotechnological applications, abiotic stress, and defense responses, are summarized.

Gene family	Coffee sp.	Number of genes/members in a family	Biological function	Reference
*AP2/ERF*	*C. arabica*; *C. canephora*; *C. eugenioides*	Putative *AP2/ERF* genes in *C. arabica*: 217; *C. canephora*: 103; and *C. eugenioides*: 133	versatile roles in development and stresses	Park et al., 2025
*MADS-box*	*C. arabica*	Putative *MADS-box* genes in *C. arabica*: 23	flower and fruit development	de Oliveira et al., 2014
*Expansin (EXP)*	*C. arabica*	Putative *EXP* genes in *C. arabica*: 11	fruit developmental/yield component	Budzinski et al., 2011
*NLR*	*C. arabica*; *C. canephora*	Putative *NLR* loci in *C. arabica*: 1311; *C. canephora*: 927	defense response	Santos et al., 2022
*NBS-LRR*	*C. arabica*; *C. canephora*	Putative *NBS-LRR* genes in the two subgenomes of *C. arabica*: 5 and 3; *C. canephora*: 4	defense response	Ribas et al., 2011
*SBP*	*C. arabica*	Putative *SBP* genes in *C. arabica*: 22	defense response/expressed in response to brown spot disease	Ren et al., 2026
*PIN*	*C. arabica*; *C. canephora*	Putative *PIN* genes in *C. arabica*: 17; *C. canephora*: 9	auxin transporter and responsive to the fungus rust	Huang et al., 2020b
*CCO*	*C. arabica*	Putative *CCO* genes in *C. arabica*: 21	carotenoid breakdown and drought stress response	Naeem et al., 2024
*U-box*	*C. canephora*	Putative *U-box* genes in *C. canephora*: 47	abiotic stress and defense response	Liu et al., 2024
*Invertases*	*C. arabica*; *C. canephora*	Putative *invertase* genes in *C. arabica*: 28; *C. canephora*: 18	abiotic stress and defense response	Ferraz et al., 2025
*SAUR*	*C. canephora*	Putative *SAUR* genes in *C. canephora*: 31	somatic embryogenesis	Zanin et al., 2022
*Gretchen Hagen 3 (GH3)*	*C. canephora*	Putative *GH3* genes in *C. canephor*a: 17	somatic embryogenesis	Pinto et al., 2019
*WOX*	*C. arabica*; *C. canephora*	Putative *WOX* genes in *C. arabica*: 23; *C. canephora*: 12	somatic embryogenesis	Dong et al., 2024
*P450*	*C. arabica*	Putative *P450* genes in *C. arabica*: 87	aroma-related compounds and coffee quality	Ivamoto et al., 2015
*Phenylalanine ammonia lyase (PAL)*	*C. canephora*	Putative *PAL* genes in *C. canephora*: 3, such as *CcPAL1, CcPAL2, CcPAL3.*	phenolic compounds and coffee quality	Lepelley et al., 2012

Similarly, gene families that are primarily involved in defense responses include *Nucleotide-binding Leucine-rich Repeat* (*NLR*) and *Nucleotide Binding Site Leucine Rich Repeat* (*NBS-LRR*). The proteins encoded by these genes act as receptors to recognize the pathogenic antigens and trigger signaling pathways. Numerous NLR loci from coffee species and a genomic locus (SH3) harboring the Resistance (*R*) genes that belong to the *NBS-LRR* gene family were identified (Ribas et al., 2011; Santos et al., 2022). The brown spot disease in *C. arabica* induced altered expression of multiple genes belonging to the *SQUAMOSA PROMOTER BINDING PROTEIN* (*SBP*) family, with remarkable changes in jasmonic acid (JA) and salicylic acid (SA) levels in *C. arabica* (Ren et al., 2026). Fungal inoculation that causes leaf rust in *C. arabica* was concordant with high expression of members of *the PIN-FORMED (PIN)* family, belonging to the auxin transporter (Huang et al., 2020b). Abiotic stress response is regulated through complex gene regulation, distinct from biotic stress, despite many features being conserved. Analysis of genes encoding *carotenoid cleavage oxygenases* (*CCOs)*, which catalyze the breakdown of carotenoids and subsequently drought stress tolerance in *C. arabica* (Naeem et al., 2024). Gene families of *invertases* that irreversibly convert sucrose to hexoses and *U-box* genes primarily involved in signal transduction were implicated in overlapping functions in abiotic stress and defense responses (Liu et al., 2024; Ferraz et al., 2025).

Somatic embryogenesis-mediated clonal propagation, germplasm preservation, and genetic transformation have attracted attention due to their biotechnological applications. In coffee, gene families belonging to the *Small Auxin-up RNA* (SAUR), *Gretchen Hagen 3* (*GH3*), and *WUSCHEL-related Homeobox* (*WOX*) were implicated in somatic embryogenesis by conjugating with hormonal signaling (Dong et al., 2024; Pinto et al., 2019; Zanin et al., 2022). In relation to coffee quality, *Cytochrome P450*, a superfamily of enzymes involved in the processing of primary/secondary metabolites, and the gene family encoding *Phenylalanine ammonia lyase* (*PAL*), which processes phenolic compounds, are promising candidates for improving coffee quality (Ivamoto et al., 2015; Lepelley et al., 2012).

## 7 Leveraging genomics and genetics resources to improve coffee yield, quality, and stress resilience

Biological processes governed by the orchestrated functions of multiple genes are regulated through a multi-layered complex regulatory network. Genetic variations across different accessions and epigenetic reprogramming, driven by developmental growth and stress responses, determine the dynamic regulation of gene expression and, subsequently, phenotypic plasticity (**Figure 2**). A few studies have identified genetic polymorphisms across different coffee species/accessions using WGS and GBS approaches. DNA polymorphism data have been used to predict phenotypes of agronomic importance, identify structural variants, and reveal SNP markers significantly associated with genes involved in coffee quality and stress responses. Notably, significantly associated SNPs related to coffee quality were found to be colocalized with genes involved in caffeine, trigonelline, 5-caffeoylquinic acid, and alkaloid trigonelline biosynthesis/pathways (Jose Luis et al., 2022; Tran et al., 2018). Likewise, significantly associated SNPs for defense response were colocalized with genes encoding *serine/threonine kinases* and *NBS-LRR* (Ariyoshi et al., 2022; Huang et al., 2020b; Mekbib et al., 2022). The selection of elite germplasms using these markers through marker-assisted selection (MAS) is an effective breeding strategy and can be applied to coffee as well. However, studies on epigenetic gene regulation in coffee remain obscured, including DNA methylation, histone modifications, and the selection of non-canonical histone variants. Although efforts have been made to identify ncRNAs involved in regulating coffee genes, their precise roles in these biological processes remain largely unresolved.

**Figure 2:**
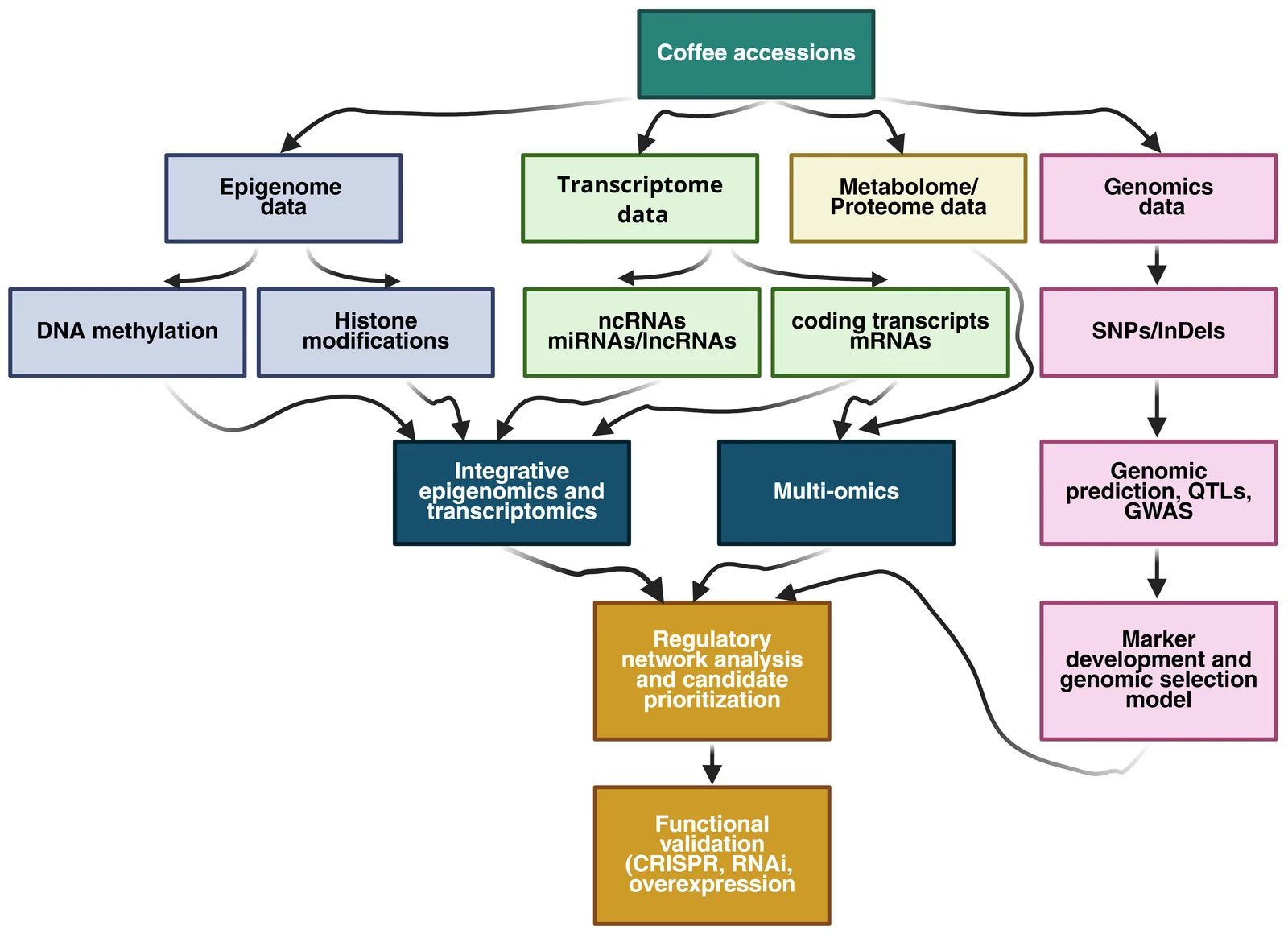
Genetic and genomic factors that govern agronomic traits. A schema of the crossing of the maternal (*C. eugenioides)* and paternal *(C. canephora)* coffee plants and the allotetraploid hybrid offspring *(C. arabica)* is shown in the top panel. The availability of high-quality, contiguous reference genomes of the two commonly cultivated coffee species (*C. arabica* and *C. canephora*) is depicted in the center panel. Various genetic and genomic elements, such as DNA polymorphisms and protein-coding and non-coding RNAs, influence coffee quality, yield, and response to abiotic and biotic stress, as depicted in the bottom panel.

Transcriptome and metabolome data are relatively abundant genomics resources of coffee. Most of these studies addressed developmental perspectives on coffee fruit/bean quality and stress responses. Metabolite profiling of coffee seeds revealed the accumulation of chlorogenic acid, cell wall polysaccharides, fatty acids, and 5-hydroxytryptamine during seed development and the deposition of a wax layer on the outer surface of coffee beans and accumulation of pheophytin in response to drought stress (**Figure 3**) (Ding et al., 2023; Joët et al., 2009). Similarly, transcriptome analysis of coffee fruits/beans revealed potential roles for several genes involved in development, coffee quality, and stress responses. Notably, expression of genes encoding *mannan synthase* (*ManS*), *galactomannan galactosyl transferase* (*GMGT*), *cell wall storage polysaccharides* (*CWSPs*) that are involved in cell wall biosynthesis/maintenance, genes encoding *PSY, β-CHY, CCD7, NCED, ABA2* that are involved in carotenoid and anthocyanin biosynthesis, genes encoding *3AT, BZ1, lcyE, MYB, NAC, bHLH* involved in fruit/bean color, genes encoding N-methyltransferases (*CaNMTs*) that catalyzes conversion of xanthosine to caffeine, genes encoding *triacylglycerol* (*TAG*) and *TAG lipase* involved in seed development, and those encoding flavonoid biosynthesis were detected (**Figure 3**) (Cheng et al., 2018; Fu et al., 2023; Hu et al., 2022; Joet et al., 2014; Pre et al., 2008; Uefuji et al., 2003; Wang et al., 2024; Zhang et al., 2022). Similarly, genes encoding raffinose biosynthesis in response to abiotic stress, genes encoding *CaSTK1, CaSAMT1, CaSLP1, CaMAS1*, *nsLTP, CaMYB1, CaERF017, CaEDR2, CaNCED, CaAPX1, CaAPX5, CaGolS3, CaDHN1*, and *CaPYL8a* for drought stress, genes encoding *RUBISCO*, *HSP*, *chlorophyll-binding protein*, and *photosystems I and II* for heat stress, and genes encoding *Receptor-like kinases* and *U-box* for defense response (**Figure 3**) were detected (de Oliveira Santos et al., 2021; Florez et al., 2019; Ivamoto et al., 2017; Liu et al., 2024; Mofatto et al., 2016; Marques et al., 2021).

**Figure 3:**
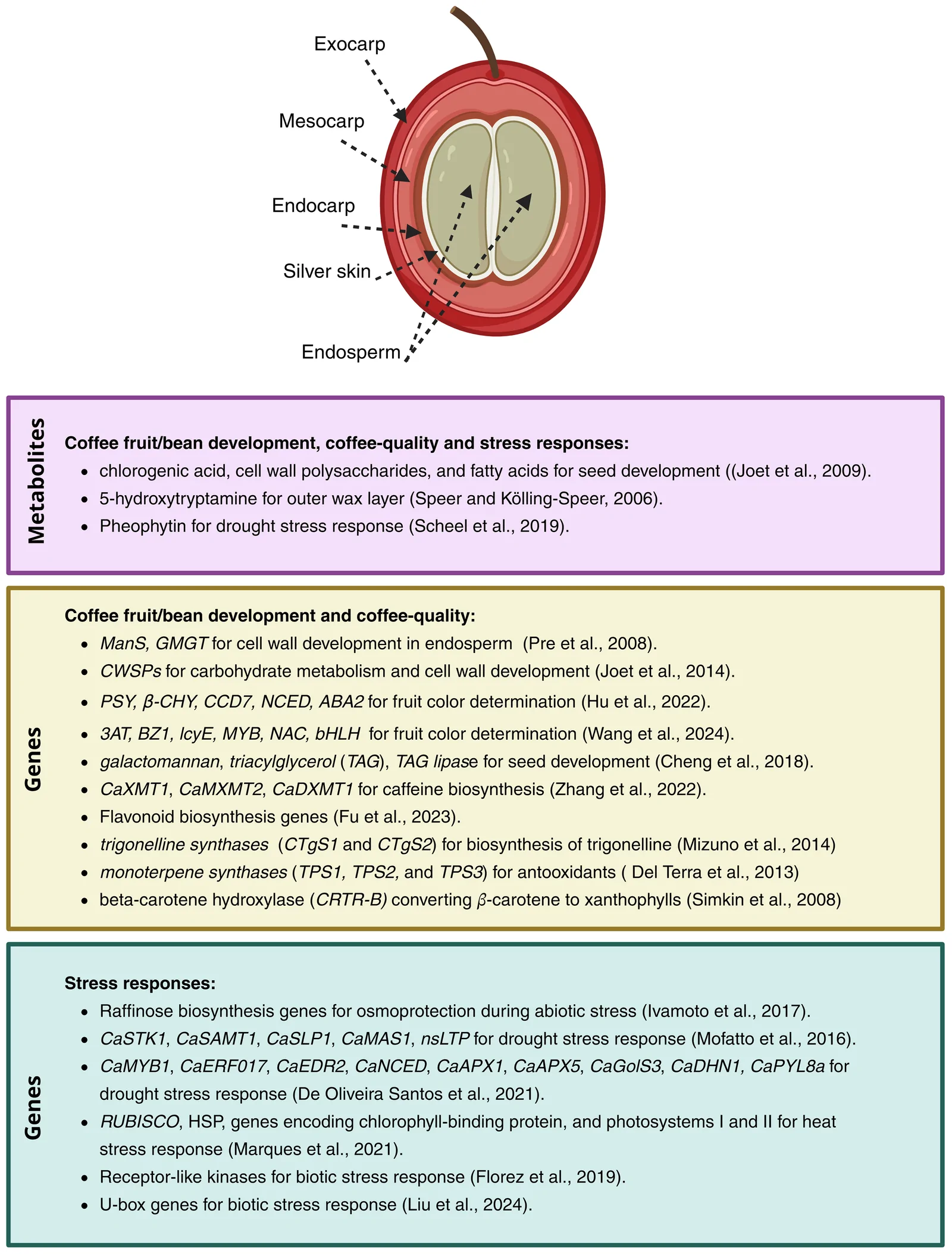
Metabolites and genes that are involved in coffee fruit/bean development and stress responses. Anatomical layers of coffee beans and key metabolites/genes involved in coffee fruit/bean development, quality, and stress responses are presented. Purple: metabolites; yellow: genes involved in development/quality; and green: genes involved in stress responses.

Additionally, a few coffee genes have been functionally characterized through reverse genetics approaches, despite being recalcitrant to conventional transformation and tissue culture techniques. The approach holds promise for meeting market demands since conventional breeding cycles are time-consuming and labor-intensive. The functions of selected coffee genes were characterized in heterologous systems. For instance, the heterologous expression of two *C. arabica* genes, presumably encoding coffee *trigonelline synthases* (*CTgS1* and *CTgS2*), in *E. coli* catalyzed the conversion of nicotinate to trigonelline (Mizuno et al., 2014). Monoterpenes are another volatile compound that contributes to coffee’s aroma. Heterologous expression of genes encoding *monoterpene synthases* (*TPS1, TPS2*, and *TPS3*) derived from *C. arabica* in *E. coli* resulted in the accumulation of terpenes and their derivatives (Del Terra et al., 2013). The catalytic activities of CaXMT1, CaMXMT2, and CaDXMT1 for the biosynthesis of caffeine were identified using heterologous expression in *E. coli* (Uefuji et al., 2003). The enzymatic activity of *C. canephora*’s *CcCRTR-B* gene that catalyzes the conversion of beta-carotene into beta-cryptoxanthin and zeaxanthin was demonstrated in *E. coli*, and a comparative analysis using chromatography revealed higher content of alpha-carotene in *C. arabica* (Simkin et al., 2008). Studies based on heterologous expression of coffee genes in *E. coli* provide useful evidence for the biochemical activity of coffee-derived proteins. However, it does not demonstrate the same gene function and activity *in planta* in coffee. Therefore, the function of those genes requires *in planta* verification by an integrative approach of genetic analysis and biochemical assays.

Further, *in planta* RNAi-mediated repression of *CaDXMT1* resulted in a substantial reduction in the accumulation of theobromine and caffeine content in embryogenic tissues and plantlets. The study established the first protocol for *Agrobacterium*-mediated transformation in coffee and developed a strategy to produce coffee with reduced caffeine content (Ogita et al., 2004). Another study revealed that silencing the gene encoding *Trehalase* in *C. arabica* using an antisense strategy enhanced tolerance to high concentrations of osmolytes, which mimic drought and/or salt stress conditions (Valencia-Lozano et al., 2025). Further improvement through precision genetic engineering via CRISPR/Cas9 demonstrated the knockout of *CcPDS* in *C. canephora*, resulting in reduced chlorophyll content (Breitler et al., 2018; Casarin et al., 2022). It shows a proof of concept for editing gene function in coffee, which is not directly relevant to coffee quality. Since the coffee quality trait is a complex blend of multiple attributes, even CRISPR/Cas9-mediated editing of a few candidate genes may not fully improve coffee. However, it provides an opportunity to improve a very specific sub-trait. For instance, a feasible strategy to knock out genes involved in caffeine biosynthesis was discussed using CRISPR/Cas9-mediated repression of the *XMT* and *DXMT* in coffee (Breitler et al., 2018; Casarin et al., 2022), potentially offering a path to developing a low-caffeine or decaffeinated coffee variety.

## 8 Limitations, knowledge gaps, and future research directions

Coffee is one of the most economically important and traded commodities. However, coffee research communities face several challenges. The remarkably narrow gene pools, due to the availability of only a few coffee species and cultivars, substantially limit breeding options. This limitation is compounded by the lack of pangenome resources in coffee, where reference genomes are primarily relied on only three species, such as *C. canephora*, *C. eugenioides*, and *C. arabica*, that represent an underutilized source of genetic polymorphisms and allelic variations. Another significant challenge is understanding the coffee-quality trait, which is a subjective and complex trait and is influenced by the balance of various metabolites, phenolics, and other compounds in specific proportions. In addition, the standardization of sensory/cupping, together with biochemical/metabolomic assessments, can serve as a complementary approach for assessing coffee cup quality. Therefore, the phenotyping of coffee quality (flavor) trait has always been challenging. The long lifespan and the many years required to reach reproductive maturity also hamper efforts to accelerate research on coffee plants. As a result, *in planta* functional verification of candidate genes is challenging and largely relies on heterologous model plants, which may not recapitulate the same gene function. Moreover, vulnerabilities to environmental extremes such as drought, heat, and pathogen invasions render outcomes unpredictable. The distinct adaptations of coffee species across different geographical altitudes and climatic conditions also contribute to these challenges. On actual agroecological farms, identifying the factors involved in adaptation to overlapping stresses/conditions remains a significant challenge. This limitation is particularly important for understanding genotype-environment interactions in the context of coffee adaptations across different geographical locations. Biotechnological applications have advanced, and new tools for genome engineering and gene function modification are available. Several caveats remain due to the recalcitrance of coffee transformation and tissue culture techniques. A few studies have conducted functional analyses of coffee genes encoding *xanthosine methyltransferases*, *monoterpene synthases*, and *trigonelline synthases* in heterologous systems (Del Terra et al., 2013; Mizuno et al., 2014; Uefuji et al., 2003). High-throughput sequencing and multi-omics-based studies have advanced for model plants and some commercially important crop plants.

Genomic resources for coffee plants have recently attracted attention, but many constraints remain due to the limited available resources. Moreover, the coffee research community is relatively small and primarily confined to a few labs. Despite many studies having been conducted to quantify transcriptomes in the contexts of coffee development, quality, and stress responses, the analyses were mostly restricted to differential gene expression and the identification of putative candidates. Actually, those complex traits are governed by the coordinated function of multiple genes through regulatory networks, which remain to be interrogated. A few studies have utilized transcriptome data to identify putative lncRNAs, as they share many similar characteristics with PCGs. However, functional verification of lncRNAs remains unexplored. Similarly, either computationally or through smRNA sequencing, coffee miRNAs and their putative target PCGs have been detected, but functional analysis remains uninterrogated. Another major gap in coffee research is the lack of foundational epigenomic resources, including DNA methylation, histone modifications, chromatin remodeling, and accessibility data. The development and quality of coffee are highly sensitive to environmental conditions. Epigenetic factors are likely to play a crucial role in adaptation and phenotypic plasticity in response to environmentally induced variation. Similarly, epigenetic studies could help clarify coffee developmental processes, including fruit development, seed maturation, and metabolic processes associated with coffee quality traits. Importantly, homeologous gene regulation in allopolyploid *C. arabica* can be explained through allele-specific expression derived from a particular parent. Such type of subgenome imbalance and expression bias (dominance/silencing) is expected to be strongly associated with differential epigenetic marks between the alleles derived from two distinct parents in *C. arabica*.

Despite these challenges, it offers an excellent opportunity for coffee research, as numerous avenues for improvement remain unresolved. The release of high-quality, contiguous reference genomes for coffee species provides a valuable opportunity to identify key genes regulating metabolites and compounds linked to coffee quality and stress resilience. This review identifies and categorizes key candidate metabolites and genes relevant to coffee improvement. In addition, with the establishment of the *Agrobacterium*-mediated transformation protocols (Breitler et al., 2018; Casarin et al., 2022; Ogita et al., 2004), genes that could be potentially involved in somatic embryogenesis (Dong et al., 2024; Pinto et al., 2019; Zanin et al., 2022), and CRISPR-Cas9 strategies to alter gene functions (Breitler et al., 2018; Casarin et al., 2022; Leibrock et al., 2022) offer unprecedented opportunities for the coffee research community. Altogether, narrow genetic diversity, limited genomics and epigenomics resources, limited success in biotechnological applications of coffee gene function testing, and plant transformation present an excellent opportunity for the coffee research community.

## 9 Conclusion

In conclusion, the genomic and functional genomics resources for coffee, including high-quality reference genomes, SNP markers, genomic prediction, metabolomes, transcriptomes, and non-coding RNAs, have been substantially expanded. These resources have been used to identify candidate genes involved in coffee quality and stress resilience. In addition, the function of a few genes has been verified in planta and/or in other heterologous systems. Despite this progress, their application to coffee breeding and improvement programs remains limited, as further validation, standardization, and testing across diverse genetic backgrounds and environments largely remains unexplored. Future progress will depend on generating additional datasets, expanding phenotyping efforts, evaluating gene functions, and translating these findings into practical solutions to improve coffee quality and stress resilience.
